# Evidence for Enhanced Mutualism Hypothesis: *Solidago canadensis* Plants from Regular Soils Perform Better

**DOI:** 10.1371/journal.pone.0015418

**Published:** 2010-11-03

**Authors:** Zhen-Kai Sun, Wei-Ming He

**Affiliations:** State Key Laboratory of Vegetation and Environmental Change, Institute of Botany, Chinese Academy of Sciences, Beijing, China; Argonne National Laboratory, United States of America

## Abstract

The important roles of plant-soil microbe interactions have been documented in exotic plant invasion, but we know very little about how soil mutualists enhance this process (i.e. enhanced mutualism hypothesis). To test this hypothesis we conducted two greenhouse experiments with *Solidago canadensis* (hereafter *Solidago*), an invasive forb from North America, and *Stipa bungeana* (hereafter *Stipa*), a native Chinese grass. In a germination experiment, we found soil microbes from the rhizospheres of *Solidago* and *Stipa* exhibited much stronger facilitative effects on emergence of *Solidago* than that of *Stipa*. In a growth and competition experiment, we found that soil microbes strongly facilitated *Solidago* to outgrow *Stipa*, and greatly increased the competitive effects of *Solidago* on *Stipa* but decreased the competitive effects of *Stipa* on *Solidago*. These findings from two experiments suggest that in situ soil microbes enhance the recruitment potential of *Solidago* and its ability to outcompete native plants, thereby providing strong evidence for the enhanced mutualism hypothesis. On the other hand, to some extent this outperformance of *Solidago* in the presence of soil microbes seems to be unbeneficial to control its rapid expansion, particularly in some ranges where this enhanced mutualism dominates over other mechanisms.

## Introduction

Plant invasion is a threat to the conservation of ecosystems and an economic problem [1], [2]. Invasive plants often perform better where they are introduced than where they are native, thereby shifting from minor components of communities at home to dominants where introduced [3]. To explain this success a variety of nonmutually exclusive hypotheses have been proposed, like enemy release, evolution of novel traits, disturbance, novel weapons, and empty niches in invaded communities [4], [5]. However, the relative importance of these hypotheses depends on the specific invasion, and the factors influencing a plant's ability to invade are not well understood [5]. Understanding the mechanisms involved in plant invasions is also required to attain biological controls.

Knowledge of the net effects of soil microbes is useful to understand their roles in determining the success of invasive plants in the real world [1], [4], [6]–[12]. However, we know very little about enhanced mutualisms in the context of invasion [but see 8], [ 13], and it is not yet understood that how interactions between plants and soil microbes influence the competitive outcomes between invasive plants and native plants. These interactions may depend on specific plants and specific soils, which in turn can alter the performance of plants [4], [9], [14]. Although how these interactions affect recruitment and competitive outcomes of invasive and native plants is very interesting, little is known about these aspects.

*Solidago canadensis* (Canada goldenrod) is an exceptionally successful worldwide invader [15], [16]. Since its introduction into China, *S. canadensis* has been spreading rapidly; despite extensive efforts to eradicate it, this species is still among the most notorious invasive plants in China [16], [17]. Despite a reduction in herbivore pressure, no indication of a rapid evolutionary shift in allocation from defence to growth due to enemy release has been observed [18]. Hence, other mechanisms are likely to be involved in the invasion success of this species. Recent studies suggest that soil microbes can enhance the successful invasion of exotic plants, such as *Acer negundo*, *A. platanoides*, *Bidens pilosa*, and *Sorghum halepense* (i.e. the enhanced mutualism hypothesis) [8], [11], [13]. To date, however, no studies have addressed how plant-soil microbe interactions affect the invasion of *S. canadensis*. Thus the purpose of this paper was to test the enhanced mutualism hypothesis by comparing the performance of *S. canadensis* between conditions with or without soil microbes. Specifically, we linked the effects of soil microbes on seedling recruitment of *S. canadensis* and its interactions with *Stipa bungeana*, a native Chinese grass, and hypothesized that soil microbes favour *S. canadensis* over *S. bungeana* in terms of emergence, growth, and competitive ability.

## Methods

### Study Species and Soils

*Solidago canadensis* (hereafter *Solidago*) L. is native to North America where it is uncommon, and is an exceptionally successful worldwide invader in Europe, large parts of Asia, Australia, and New Zealand [15], [16]. *Solidago* often invades roadsides, abandoned fields, agricultural fields, and pastures in China [17]. Due to its fast growth, prolific reproduction, and strong allelopathic effects on native plant species, *Solidago* can shape near monocultures in its introduced range [17]. *Stipa bungeana* (hereafter *Stipa*) Trin. is native to China, Mongolia, and Japan, and widely distributed across China [19]. *Stipa* is the most dominant grass in some steppe ecosystems [20], [21]. In the field, *Solidago* can replace *Stipa* and shape near *Solidago* monocultures. We chose *Stipa* as a model native plant because it is highly typical and dominant in local plant communities. Previous studies imply that *Solidago* and *Stipa* may have AM fungi [22], [23], and the former often destroys local ecosystems and the latter can conserve degraded ecosystems. Seeds of *Solidago* were collected from its monocultures, and seeds of *Stipa* were collected from the grasslands where *Stipa* dominates but *Solidago* did not invade.

Since plant-soil microbe interactions depend on the specific plants and soils [4], [9], [14], we only collected soils from the rhizospheres of *Solidago* and *Stipa* in the two types of plant communities aforementioned. *Solidago* soil can indicate how the soil pre-cultured by *Solidago* affects itself and native plants, and the *Stipa* soil can provide insights into why *Solidago* is able to invade successfully. We located 20 sampling sites in each plant community, which were about 10 meters apart. Specifically, we chose 20 similar-sized mature plants of *Solidago* and *Stipa* from a *Solidago* monoculture and *Stipa* grassland, respectively, and then collected soils from the rhizospheres of *Solidago* and *Stipa*. All soil samples were air-dried and then sieved with a 2 mm sieve. Since soil is highly heterogeneous, that is, soil traits greatly vary in space [24], [25], 20 soil samples from a *Solidago* or *Stipa* community were completely composited to homogenize initial soils. Finally, the homogenized soils were separated into two portions for the sterilized and control treatments. For the sterilized soils, they were treated by autoclaving (120°C, 30 min) on three consecutive days to kill soil microbes. This approach has been widely used in related studies [1], [4], [11]. For the control soils, they were kept intact. Sterilized and non-sterilized soils were filled into 40 Petri dishes for experiment 1 and 120 pots for experiment 2.

### Experiment 1: Seedling Emergence

A greenhouse experiment was carried out at the Institute of Botany of Chinese Academy of Sciences (IBCAS) with five replicates of 50 seeds per treatment. Specifically, seeds of each species were placed in 9 cm Petri dishes filled with sterile or non-sterile soils of 1 cm depth. Greenhouse temperatures, relative humidity, and photosynthetically active radiation during the day were 20–25°C, 50–60%, and above 1200 µmol m^−2^ s^−1^. All dishes were watered as needed to maintain adequate soil moisture. Emergence was checked daily and then seedlings were removed. This experiment lasted for 30 d from 15 March to 14 April 2010. Emergence was assessed on five dishes per treatment.

The experimental design involved a factorial analysis of variance with three factors (i.e. sterilization, species identity, and soil source), each with two levels. Three-way ANOVAs were used to test the effects of sterilization (sterile versus non-sterile), species identity (native versus exotic), soil sources (*Solidago* rhizosphere versus *Stipa* rhizosphere), and their interactions on emergence. One-way ANOVA also was used to test the effects of a single factor. All the statistical analyses were carried out using SPSS 13.0 (SPSS Inc., Chicago).

### Experiment 2: Growth and Competitive Ability

We conducted a second experiment in the same greenhouse as in experiment 1 at the IBCAS, in which plants of *Solidago* and *Stipa* were grown alone or plants of *Solidago* were planted in competition with *Stipa*. In this experiment all plants were grown from seeds in 250 ml pots filled with sterile or non-sterile soils from the rhizospheres of *Solidago* and *Stipa*. Plants were supplied with 20 ml of water at 1–3 d intervals, depending on how fast the soil dried. No nutrients were added during the experiment. Greenhouse temperatures, humidity, and lighting were described above. Each combination includes 10 replicates. This experiment ran from 18 March 2010 to 28 June 2010. At the end of the experiment, all plants were harvested, washed, dried at 60°C for 72 h, and then weighed.

To quantify competitive effects, relative interaction intensity (RII) was calculated as follows:
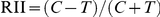
where *C* is the biomass of plants grown with a neighbor and *T* is the biomass of plants grown alone [26]. RII has values ranging from 1 to −1, is symmetrical around zero, and is negative for competition and positive for facilitation [26].

The experimental design involved a factorial analysis of variance with three factors (i.e. sterilization, species identity, and soil source), each with two levels. Three-way ANOVAs were used to test the effects of sterilization (sterile versus non-sterile), species identity (native versus exotic), soil sources (*Solidago* rhizosphere versus *Stipa* rhizosphere), and their interactions on the total biomass per plant and competitive effects. One-way ANOVA also was used to test the effects of a single factor. All the statistical analyses were carried out using SPSS 13.0 (SPSS Inc., Chicago).

## Results

**Figure 1:**
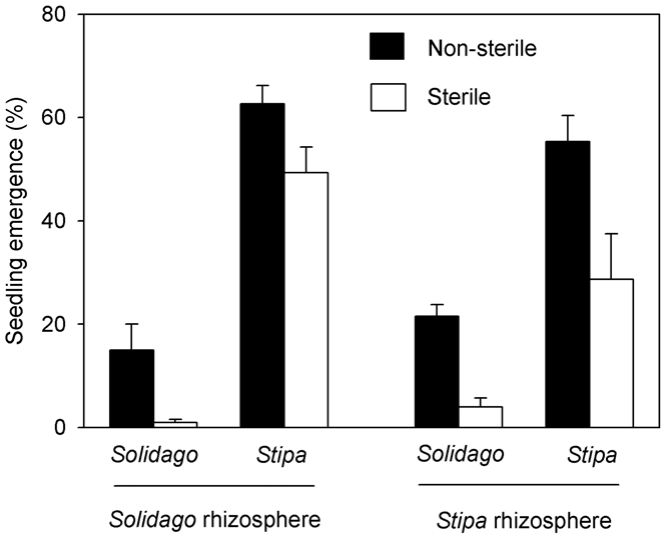
Seedling emergence (means +1 SE, n = 5) under eight different combinations consisting of sterilization, species identity, and soil source. See Table 1 for ANOVAs.

Emergence of both *Solidago* seeds and *Stipa* seeds was higher in non-sterile soils than sterile soils (*P*<0.05, Fig. 1; Table 1), suggesting soil microbes enhance the seeds to emerge. Interestingly, this facilitative effect was much stronger in *Solidago* than *Stipa* (Fig. 1). For example, in the *Solidago* soil sterilization decreased emergence by 93% and 22% for *Solidago* and *Stipa*, and in the *Stipa* soil sterilization decreased emergence by 81% and 48% for *Solidago* and *Stipa*. Emergence was lower in *Solidago* than *Stipa* (*F* = 136.1, *P*<0.0001, Table 1), particularly in the *Solidago* soil (*F*_species identity × soil source_  = 8.02, *P* = 0.008, Table 1).

**Table 1:** Three-way ANOVAs for the effects of sterilization, species identity (SI), soil source (SS), and their interactions on seedling emergence, total biomass per plant, and relative interaction intensity.

	Seedling emergence			Total biomass per plant			Relative interaction intensity		
Effect	df	*F*	*P*	df	*F*	*P*	df	*F*	*P*
Sterilization (S)	1	29.15	**<0.0001**	1	440.5	**<0.0001**	1	5.22	**0.026**
Species identity (SI)	1	136.1	**<0.0001**	1	49.81	**<0.0001**	1	11.33	**0.001**
Soil source (SS)	1	1.95	0.172	1	248.7	**<0.0001**	1	8.67	**0.005**
S×SI	1	0.41	0.526	1	141.2	**<0.0001**	1	32.38	**<0.0001**
S×SS	1	1.62	0.213	1	96.87	**<0.0001**	1	4.17	**0.045**
SI×SS	1	8.02	**0.008**	1	15.99	**<0.0001**	1	0.07	0.798
S×SI×SS	1	0.55	0.463	1	0.314	0.577	1	0.002	0.963

**Figure 2:**
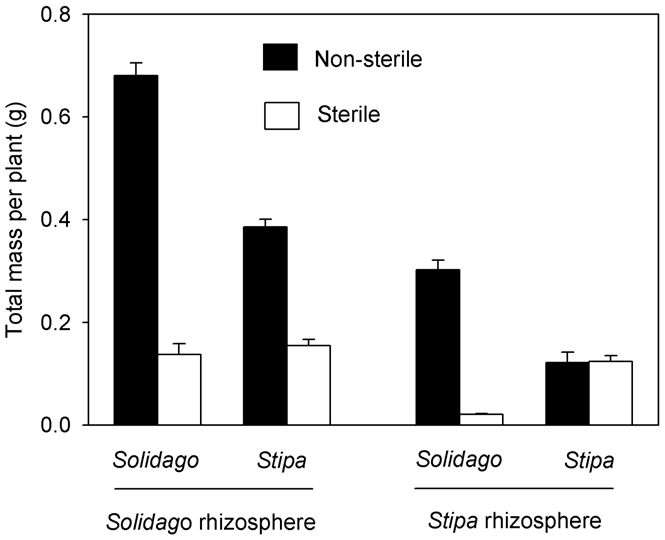
Total dry biomass per plant (means +1 SE, n = 10) under eight different combinations consisting of sterilization, species identity, and soil source. See Table 1 for ANOVAs.

Sterilization, species identity, soil source, and their interactions (*P*<0.0001, Table 1), except for the three-factor interaction (*F* = 0.314, *P* = 0.577, Table 1), affected the final dry total biomass. The total biomass of *Solidago* was 76% higher than that of *Stipa* when the *Solidago* soil was kept intact (*F* = 46.79, *P*<0.0001, Fig. 2); however, *Solidago* plants and *Stipa* plants shared equal biomass when the *Solidago* soil was sterilized (*F* = 0.534, *P* = 0.879, Fig. 2). For the *Stipa* soil, *Solidago* had greater biomass than *Stipa* in the presence of soil microbes (*F* = 26.39, *P*<0.0001) and the opposite was true in the absence of soil microbes (*F* = 24.84, *P*<0.0001) (Fig. 2). Overall the total biomass of *Solidago* was greater than that of *Stipa* in the presence of soil microbes across two soils (*F* = 17.361, *P*<0.0001) and the opposite was the case in the absence of soil microbes (*F* = 7.544, *P* = 0.010) (Fig. 2). Thus in situ soil microbes in the introduced range were able to help *Solidago* outgrow *Stipa*.

**Figure 3:**
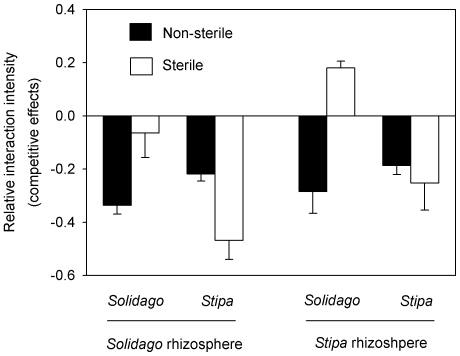
Competitive effects as indicated by relative interaction intensity (means +1 SE, n = 10) under eight different combinations consisting of sterilization, species identity, and soil source. See Table 1 for ANOVAs.

Competitive effects, as indicated by relative interaction intensity, were affected by sterilization, species identity, soil source, and the interactions of sterilization with species identity or soil source (*P*<0.05, Table 1). Competitive effects of *Solidago* on *Stipa* were significantly stronger in the presence of soil microbes than in the absence of soil microbes, regardless of in the *Solidago* soil (*F* = 7.727, *P* = 0.012) or *Stipa* soil (*F* = 29.167, *P*<0.0001) (Fig. 3). Conversely, competitive effects of *Stipa* on *Solidago* were much weaker in the non-sterile *Solidago* soil than the sterile *Solidago* soil (*F* = 10.920, *P* = 0.004), and similar between non-sterile and sterile *Stipa* soil (*F* = 0.372, *P* = 0.552) (Fig. 3). Most importantly, *Solidago* had stronger competitive effects than *Stipa* in the presence of soil microbes across two soil sources (*F* = 5.860, *P* = 0.021) and the opposite was true in the absence of soil microbes (*F* = 23.076, *P*<0.0001) (Fig. 3). Thus competitive advantages of *Solidago* were greatly enhanced by soil microbes in its introduced range.

## Discussion

Our results that soil microbes significantly enhanced emergence, growth, and competitive ability of *Solidago* provide strong evidence for the enhanced mutualism hypothesis. Meanwhile these findings also suggest that the successful invasion of *Solidago* can in part be attributable to in situ soil microbes in its introduced range. Previous studies have indicated that invaders commonly escape inhibitory soil biota, but unlike our findings that soil biota enhance its growth. For example, Kulmatiski et al. found for invaders in their introduced ranges soil biota effects were not positive, but either very weak or neutral, but these invaders had more negative feedbacks in their native range [9]. Callaway et al. found that sterilization effects were stronger from soils collected in the native range of an invader than soils collected in the non-native range, and that positive feedback occurred in the soils from the non-native range and negative feedback did in soils from the native range [1]. A recent study proposed that negative soil feedback relationships accumulate over time for exotics [27]. This needs to be further examined for *Solidago*.

In our experiments AM fungi conferred disproportional effects on emergence, growth, and competitive ability of both *Solidago* and *Stipa*. That is, *Solidago* obtained more benefits from AM fungi than *Stipa*. One possibility is that there is a shift in the continuum of parasitism to mutualism that plants and mycorhizae have. A second possibility is that there is an evolution towards stronger parasitism in the native range whereas this evolution does not occur in the non-native range. Accordingly, soil mutualists may be stronger for invasive species than native species, so are they in one range than another. This phenomenon could be true for other soil biota as well. It is most likely that there are different ways by which AM fungi enhance *Solidago*'s performance. For example, emergence may benefit from fungi that provide the germinating seeds with carbon and nutrients [14], [28], the growth of plants may be enhanced through root-fungus mutualisms or nutrient uptake [1], [12], [29], and mycorrhizae may differentially alter the growth of plants and their tolerance to herbivore [30]. Additionally, mycorrhizal densities also contribute to plant invasions [31].

It is already known that allelopathy is another mechanism by which *S. canadensis* invades successfully. Specifically, allelopathic exudates from the roots or leaves of *S. canadensis* severely inhibit the growth of native Chinese plants, thereby greatly contributing to its successful invasion [17], [32]. In a recent study allelopathic compounds in *S. canadensis* strongly restrain the native European flora [33]. Such allelopathic effects have been repeatedly reported in other notorious invaders like *Centaurea maculosa* [34]–[36]. Most interestingly, *Solidago-*soil microbe interactions can alter allelopathic effects of *Solidago* [33]. Additionally, root exudates from the invader *Chromolaena odorata* stimulated the abundance of the soil pathogen *Fusarium spp*, thereby reducing seedling growth of native species [37]. Thus, the joint roles by soil microbes and allelopathy of *Solidago* await further study.

Inhibitory and beneficial effects of soil microbes on plants depend on the net effect of accumulating pathogenic and mutualistic soil organisms, and these feedbacks may alter plant-soil microbe interactions in ways that may facilitate invasion and inhibit re-establishment by native species [4]. For example, the invasive plant *Chromolaena odorata* accumulates soil pathogens which inhibit native plants [37]. Plants grown in soil pre-cultivated by individuals of the same species often show reduced performance, commonly attributed to the accumulation of soil biota that have an inhibitory effect on subsequent plant growth [27]. Interestingly, we found something else for both *Solidago* and *Stipa*. For example, emergence and biomass of the native plant Stipa were greater in the non-sterile *Solidago* soil than in sterile *Solidago* soil, and both *Solidago* and Stipa did not reduce their emergence and total biomass in the presence of soil microbes. Consequently, these findings suggest that the net effects of plant-soil microbe interactions may be positive or neutral, but negative for these two species.

*Solidago* and *Centaurea* belong to Asteraceae, but both invaders exhibit contrasting responses to mycorrhizae. *Centaurea maculosa* plants grown alone were 50% smaller in the presence of soil microbes than in the absence of soil microbes [38] and mycorrhizae had no direct effect on the growth of *C. maculosa* and the native plant *Festuca idahoensis* [39]. In contrast, soil microbes from the *Solidago* rhizosphere enhanced the growth and competitive effects of *Solidago* simultaneously, and in the presence of soil microbes *Solidago* exhibited higher growth and competitive advantages relative to *Stipa*. Consequently, in situ soil microbes help *Solidago* outcompete *Stipa* and become the final winner via faster growth and stronger competitive ability, regardless of in naïve plant communities or those communities dominated by *Solidago*. Soil microbes are important in determining the outcomes of interspecific competition [40]. For example, fungi increased *C. maculosa's* negative effect on North American natives [39], [41]. Competitive interactions of the invasive shrub *Ardisia crenata* with the native plant *Prunus caroliniana* depended on the isolates of mycorrhizae present [42].

To date we know very little about how plant-soil microbe interactions affect seedling recruitment of invasive plants, though this process determines population dynamics and community development [43], [44]. Sterilization dramatically decreased emergence of *Solidago* and *Stipa*, indicating that soil microbes strongly enhance their recruitment potential through increasing emergence. Due to its extremely tiny seeds, *Solidago* plants often produce huge numbers of seeds; meanwhile, they have profuse rhizomes that can yield a lot of clonal ramets [17]. This phenomenon does not occur in *Stipa*. Thus, it is most likely that *Solidago* plants have the higher potential to recruit due to prolific sexual and asexual reproduction, and facilitative effects by in situ soil microbes.

In summary, these findings suggest that the recruitment potential and competitive advantages of *Solidago* can be enhanced by soil microbes in its introduced range, thus support the enhanced mutualism hypothesis. The invasion success of *Solidago* in China can in part be attributable to in situ soil microbes. There may be a variety of different mechanisms jointly driving the success of *Solidago*; however, little is known about the relative importance of these mechanisms. It is likely that to some extent this outperformance of *Solidago* in the presence of soil microbes may be unbeneficial to control its rapid expansion, particularly in some ranges where the enhanced mutualism dominates over others.
